# Colored LED Lights: Use One Color Alone or with Others for Growth in *Hedyotis corymbosa* In Vitro?

**DOI:** 10.3390/plants12010093

**Published:** 2022-12-24

**Authors:** Anh Tuan Le, In-Lee Choi, Gyung-Deok Han, Ho-Min Kang, Dae Ho Jung, Won-Pyo Park, Mehtap Yildiz, Thuong Kiet Do, Yong Suk Chung

**Affiliations:** 1 Department of Plant Resources and Environment, Jeju National University, Jeju 63243, Republic of Korea; 2 Department of Plant Physiology, Faculty of Biology—Biotechnology, University of Sciences, VNU-HCM, Ho Chi Minh 70000, Vietnam; 3 Agricultural and Life Science Research Institute, Kangwon National University, Chuncheon 24341, Republic of Korea; 4 Department of Practical Arts Education, Cheongju National University of Education, Cheongju 28690, Republic of Korea; 5 Interdisciplinary Program in Smart Agriculture, Kangwon National University, Chuncheon 24341, Republic of Korea; 6 Division of Smart Horticulture, Yonam College, Cheonan 31005, Republic of Korea; 7 Department of Agricultural Biotechnology, Faculty of Agriculture, Van Yuzuncu Yil University, Van 65080, Turkey

**Keywords:** chlorophyll-a fluorescence, *Hedyotis corymbosa*, light-emitting diode (LED), photosynthesis, total triterpenoid

## Abstract

In recent years, light-emitting diode (LED) technology has been applied to improve crop production and induce targeted biochemical or physiological responses in plants. This study investigated the effect of different ratios of blue 450 nm and red 660 nm LEDs on the overall plant growth, photosynthetic characteristics, and total triterpenoid production in the leaves of *Hedyotis corymbosa* in vitro plants. The results showed that a high proportion of blue LED lights had a positive effect on enhancing photosynthesis and the overall biomass. In addition, blue LED lights were shown to be more effective in controlling the production of the total triterpenoid content compared with the red LED lights. Moreover, it was also found that plants grown under a high proportion of red LEDs exhibited reduced photosynthetic properties and even induced damage to the photosynthetic apparatus, which indicated that the blue or red LED lights played contrary roles in *Hedyotis corymbosa*.

## 1. Introduction

Along with an increasing population and climate change, the world is facing problems related to safe agriculture, the environment, and limited resources of medicinal plants, which requires the producer to have to choose the appropriate planting variety and cultivation method [1]. Using artificial light sources is more useful than using chemicals to control the seed quality, flowering, yield, and diseases in the crops and simultaneously reduce the environmental impact. Consequently, the use of LED lights in agricultural farming has been increasingly noticed [2,3,4,5]. Several studies have shown that changes in LED wavelengths and light intensity could be the tools to manipulate plant growth, photosynthesis performance, and plant secondary metabolism pathways for the production of functionalized foods [5,6,7,8].

The most important light wavelengths for photosynthesis, which are the energy source to create the secondary compounds, are in the blue and red regions [9,10]. Therefore, there are numerous research efforts on the effects of blue and red light [5,7,8]. The light wavelength, via the plant photoreceptor, is an important environmental factor for the regulation of photosynthesis and the photomorphogenesis of plants, including shoot elongation, antioxidant enzyme activities, leaf anatomy, chlorophyll content, and chlorophyll fluorescence [2,11,12,13]. Red light triggers the photosynthetic process [14]; blue light enhances the photosynthetic activity [15,16]. A trend of an increase in the biomass and second metabolic production was demonstrated when a combination of red and blue LEDs was used [7,14,17,18]. Although there are a few studies using a ratio (such as on *Vanilla planifolia Jacks* [19], *Myrtus communis* L. [18], and *Gerbera jamesonii* [14]), the number of studies is small and the target effects are limited.

Triterpenoid is one of the largest groups of secondary metabolites, with a diverse biological activity in herbal plants. It is abundant in *H. corymbosa*, which is a popular weedy herb that is easy to find and be cultivated in Vietnam, India, China, America, and Africa; therefore, it has great potential to be used as a triterpenoid resource for food and medicine [20]. It includes ursolic acid (3β-hydroxyurs-12-en-28-oic acid, UA) and oleanolic acid (3β-hydroxyolean-12-en-28-oic acid, OA) [21,22,23]. These compounds are mainly responsible for the pharmacological activity of this plant in hepatoprotective, anticancer, and spasmolytic activities; they are antipruritic, antiallergic, and antibacterial and they have antitumor and antioxidant properties [23,24,25,26]. However, the raw materials collected in the wild are heterogeneous, have a high post-harvest cost, and mainly depend on environmental conditions. According to previous studies, a change in the light quality could lead to increased levels of the total phenolic and flavonoid compounds in *H. corymbosa* [27,28,29].

Thus, the current study was designed to determine the effect of overall plant growth, the chlorophyll fluorescence parameters, and the photosynthetic characteristics of the leaves of *H. corymbosa* using not only the effect of blue and red lights, but also different ratios of blue:red LEDs on one of the most important secondary metabolites, triterpenoid.

## 2. Results

Five different LED ratios were used and were compared with a fluorescent lamp (FL) as the control to evaluate the chlorophyll fluorescence parameters in the leaves of *H. corymbosa* in vitro (Table 1). The values of F_v_/F_m_, Y(II), qP, and ETR in the leaves under 100% or 70% red LEDs were lower than in other treatments whereas the Y(NO) values under those treatments were higher. There were no differences between all the LED treatments and the Y(NPQ) and qN values. Monochromatic LEDs reduced the F_m_ value compared with the other LED combinations. The F_0_ value of the leaves under the combination of the B3R7 LEDs was higher; conversely, this value of the leaves in the blue LED treatment was lower than the control (data not shown). The plants grown under a high proportion of blue LEDs had an F_v_/F_m_ value higher than 0.7 whereas the treatment with high red LED ratios (100% or 70% red LEDs) had values lower than 0.7. Notably, the value of ETR under the blue LED lights was almost 1.5-fold higher than that under the red LED light. Various studies have demonstrated that a long-term red LED treatment reduces the quantum yield of PSII photochemistry; blue or BR-combined LEDs could alleviate those symptoms [15,17,30,31]. However, our study showed that the plants under high red LED ratios (B3R7) also responded with the same trend as the red LEDs and a high proportion of blue LEDs was more effective on the photosynthesis properties of *H. corymbosa* in vitro.

**Figure 1:**
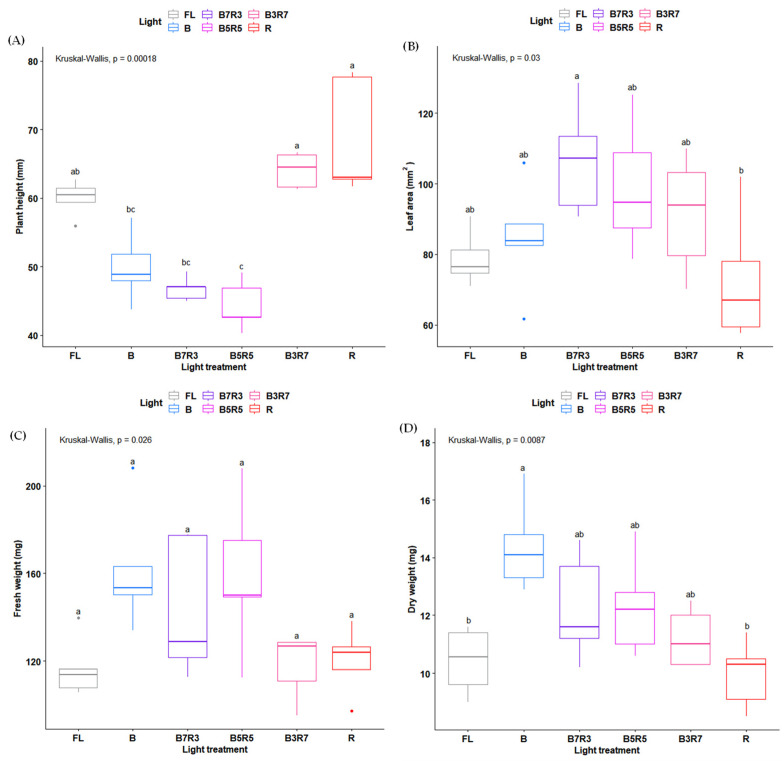
Growth parameters, including plant height (**A**), leaf area (**B**), fresh weight (**C**), and dry weight (**D**), of *H. corymbosa* in vitro after 4 weeks under different light treatments at the same PPFD of 50 µmol·m^−2^·s^−1^. Statistical analysis was performed with a Kruskal–Wallis test followed by a Dunn–Benjamini–Hochberg post hoc test for multiple comparisons. Different letters indicate significant differences between two medians.

The change in different LED light ratios led to significant impacts on the plant morphogenesis and biomass of *H. corymbosa* in vitro (Figure 1). Compared with all light treatment experiments, a high ratio of red LEDs increased the plant height whereas the plants that were treated with a high blue LED ratio decreased that trait (Figure 1A). The leaf area was larger in plants under the three combinations of the blue and red LEDs and lower in the plants grown under monochromatic red LEDs compared with the control light (Figure 1B). Monochromatic blue LEDs were more effective on the biomass compared with the red, B3R7 LEDs, and control light treatments. Most studies have demonstrated that BR-combined LED lights contribute to an enhanced overall plant biomass compared with other light treatments [14,18,19,32,33]; however, it was not applicable for *H. corymbosa* in vitro. There was no fresh and dry weight difference measured between all combinations of the BR combination LEDs and the control light (Figure 1C,D). Therefore, monochromatic blue and red were chosen for further experiments to establish which color was more effective in photosynthesis and the triterpenoid content.

The monochromatic LEDs caused a significant decrease in the SPAD values in the leaves of *H. corymbosa* in vitro compared with the white fluorescent lamps; the lowest value was observed in the red LED treatment (Table 2). The SPAD value was measured based on the chlorophyll optical properties, which are correlated to the total chlorophyll (a + b) in a leaf [34,35]. Our results suggested that the monochromatic light reduced the photosynthetic pigment concentration compared with the control. However, only the red LEDs depressed the evolution of oxygen in the light stage, which was presented by Hill reaction activity lower than the control. The plants under the blue LEDs did not have this expression (Table 2).

Table 3 shows the significant difference in the triterpenoid production involved in the plant growth under different light sources. The total triterpenoid content of the plants with the blue LED treatment was almost 1.4-fold higher than that under the red LED lights, which was observed via a spectrophotometric method (Table 3).

## 3. Discussion

The monochromatic LED light caused a reduction in the photosynthetic pigments in the leaves of *H. corymbosa* in vitro (Table 2). Blue LEDs, which have a short wavelength and a high energy light [36], played as a strong light environment condition that caused a decrease in the LHC amount and chlorophyll-b biosynthesis from chlorophyll-a through the enzyme chlorophyllide, an oxygenase [37]. A reduction in the chlorophyll-b content in the leaves of ex vitro plants grown under blue LEDs compared with a control was previously reported [27,28]. Plants that were exposed to a long-term red LED light (660 nm) treatment showed “red light syndrome”, which had a lower chlorophyll-a concentration in the leaves compared with the control in cucumbers and lettuce [12,31]. Furthermore, a high proportion of red LEDs caused a strong reduction in the activity of chlorophyll-a in the PSII centers; this was indicated by a lower maximal quantum yield of PSII photochemistry (F_v_/F_m_) in dark-adapted leaves (Table 1). Research on Arabidopsis showed that red lights significantly affected D1-protein degradation, which plays an important role in maintaining the photosynthetic activity and functional PSII [38]. Our F_v_/F_m_ values below 0.7 reflected the damage to PSII [23], which led to a decrease in the Hill reaction activity of the chloroplast suspension isolated from the leaves under the red LED treatment compared with the plants under the blue LEDs and the control (Table 2).

In the light-adapted measurement, when the leaves were exposed to a continuous treatment light, the quantum yields of the photochemical and non-photochemical energy conversion in PSII were calculated and displayed as the parameters Y(II), Y(NPQ), and Y(NO) [39,40]. We considered that a high blue LED proportion maintained the F_v_/F_m_ values of PSII, thus increasing the energy distribution for photochemical quenching, i.e., Y(II). The lower F_v_/F_m_ values of the PSII reaction centers in the plants grown under 70% or 100% of red LEDs led to a decrease in the Y(II) value (Table 1). On the other hand, the quantum yield of the non-regulated heat dissipation and fluorescence emission, Y(NO), was significantly higher in the plants under a high red LED proportion compared with the control. The qN and Y(NPQ), which reflect the photoinactivation of PSII and zeaxanthin-related mechanism of excitation energy release [39,41], were not different between any of the light treatments. The chlorophyll fluorescence and heat loss were in direct competition with the processes of photochemistry for excitation energy, which is always unity: Y(II) + Y(NPQ) + Y(NO) = 1 [40]. The quantum yield of the PSII photochemistry and qP was a direct linear relationship with the electron transport rate through PSII (ETR) and related to the quantum yield of CO_2_ assimilation by the leaf [14,39,40]. Our results suggested that blue LEDs could enhance the PSII electron transfer system and use it for the photochemical pathways. On the opposite, the plants under the red LEDs distributed more energy for the exothermic processes and reduced the photochemistry.

The light energy via the electron flow along the electron transport chain was converted to ATP and NADPH. These molecules fuel the Calvin–Benson cycle for CO_2_ fixation to produce carbohydrates and other assimilatory processes [42]. An increase in the photosynthetic product accumulation (such as sugar or starch) led to the rise of the fresh and dry weight of the leaf of *H. corymbosa* [27,28]. Plants grown under a high proportion of blue LEDs had greater F_v_/F_m_, Y(II), qP, and ETR values compared with the red LEDs, which correlated with a high biomass under those light treatments [30]. Furthermore, the different light wavelengths were demonstrated to regulate the terpenoid production in the plants through photoreceptors such as cryptochromes or phytochromes [5,8]. Research on white birch reported that blue LEDs had a significant potential to increase triterpenoid production compared with red LEDs [43]. The differentially expressed genes (DEGs) analysis indicated that blue lights upregulated the expression genes of mevalonic acid pathways such as squalene synthase (SQS), squalene monooxygenase or epoxidase (SQE), and oxidosqualene cyclases (OSCs) whereas those genes under red lights were downregulated [44,45]. Our study provided evidence that blue LEDs could be more effective on triterpenoid accumulation in *H. corymbosa* in vitro.

## 4. Materials and Methods

### 4.1. Plant Material and Growth Conditions

*H. corymbosa* seeds were harvested from plants in their natural habitats. These seeds were sown in 500 mL glass culture vessels containing a 75 mL Murashige and Skoog medium with a pH of 5.8, 30 g/L sugar, and 6 g/L agar [46]. The in vitro plants, which were 10 days old after germinating, were transferred to the light treatment. The culture conditions were controlled at 27 ± 2 °C with a relative humidity of 65 ± 5% under a white fluorescent lamp and a 12 h of light and 12 h of dark (12/12) photoperiod. The photosynthesis photon flux density (PPFD) was controlled at 50 μmol·m^−2^·s^−1^ and measured by LI-250A with a LI-190R Quantum Sensor (LI-COR Inc., Lincoln, NE, USA).

### 4.2. Light Treatment

The in vitro plant cultures were incubated in a growth chamber for 4 weeks under the 6 following light sources, with a 12/12 photoperiod and at the same PPFD of 50 μmol·m^−2^·s^−1^:

1. White fluorescent lamp (FL);
2. 100% blue (B);
3. 70% blue and 30% red (B7R3);
4. 50% blue and 50% red (B5R5);
5. 30% blue and 70% red (B3R7);
6. 100% red (R).

**Figure 2:**
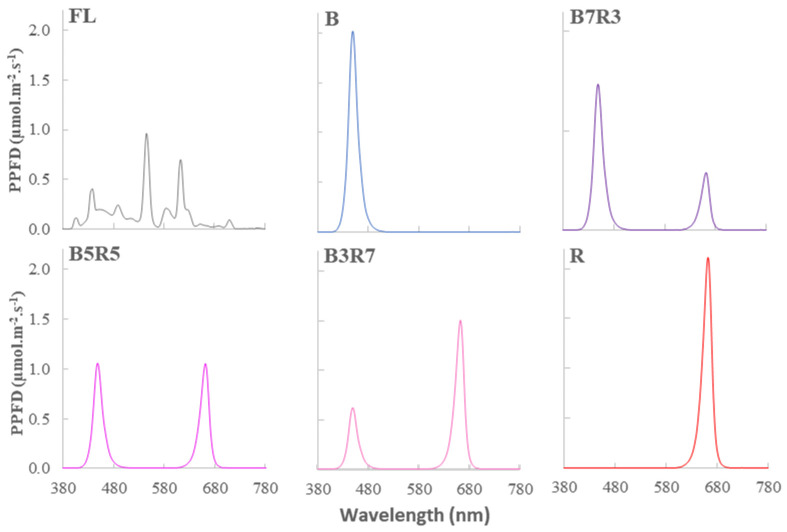
The spectra of photosynthesis photon flux densities of light treatments used in this study. All light treatments were at the same PPFD of 50 μmol·m^−2^·s^−1^.

The LED light tubes were provided by Smart Agriculture Lighting Technology, Republic of Korea. The emission spectra from the light sources were measured with an MK-350S (UPRtek, Taipei, Taiwan) (Figure 2).

### 4.3. Measurements

The measurements of the chlorophyll fluorescence parameters were obtained as follows. The fifth leaf (from the top) of *H. corymbosa* was isolated and dark-adapted for at least 15 min before the measurement. The leaf sample was recorded for the minimum fluorescence (F_o_) under modulated light (0.1 μmol·m^−2^·s^−1^); subsequently, the maximum fluorescence yield in the dark-adapted samples was measured (F_m_) and determined after a light-saturating pulse of about 5700 μmol·m^−2^·s^−1^ with an 0.8 s duration by a red light (660 nm). These leaves were light-adapted for 10 min with an actinic light (similar to the growing light conditions) and then the maximum fluorescence value (F_m_’) after a saturation pulse of red light (5700 μmol·m^−2^·s^−1^; 0.8 s duration) and the minimum fluorescence value (F_o_’) in 5 s of far-red light were automatically recorded following the program of a PAM-2500 portable chlorophyll fluorometer (Heinz Walz GmbH, Effeltrich, Germany). The parameters of the chlorophyll fluorescence included the F_v_/F_m_ maximal quantum yield of photosystem II (PSII; from 0 to 1); Y(II), the effective photochemical quantum yield of PSII; Y(NO), the quantum yield of the non-regulated heat dissipation and fluorescence emission; Y(NPQ), the quantum yield of the downregulatory non-photochemical quenching; qP, the photochemical fluorescence quenching coefficient; qN; the non-photochemical fluorescence quenching coefficient; and ETR, the relative electron transfer rate, which was calculated according to the manual and the work of Baker [39].

The SPAD (soil plant analysis development) value of the leaves was measured by a SPAD 502 Plus Chlorophyll Meter (Konica Minolta Sensing, Inc., Sakai, Osaka, Japan). The results of the measurement were based on the transmission rate of light at two wavelengths, red (650 nm) and near-infrared (940 nm), through the leaves of the plant [34].

The isolation of the chloroplasts and the determination of the Hill reaction activity was achieved as described by Henselová et al. with slight modifications [47]. To isolate the chloroplasts, 0.5 g of mature leaves was ground in a mixture of 9 mL NaCl 0.35 M and 1 mL Tris 50 mM with a pH of 8. The extract mixture was centrifuged at 500 rpm (5 min) and the supernatant was collected. The supernatant was further centrifuged for a second time at 2000 rpm (5 min) and the residue collected contained the isolated chloroplasts. The manipulations were performed at 3−5 °C in the dark. The chloroplast density was determined using a Neurban erythrocyte counting chamber. A total of 0.5 mL of the chloroplast suspension was mixed with 0.5 mL of a phosphate buffer, which consisted of 0.15 M Na_2_HPO_4_·12 H_2_O and 0.15 M KH_2_PO_4_ (8:2) (pH 6.5), and 0.125 mL of 2,6-dichlorophenol indophenol 0.25 × 10^–4^ M (DCIP). The Hill reaction activity was determined through the color loss of DCIP by optical density at 600 nm (GENESYS™ 30, Thermo Fisher Scientific Inc., Waltham, MA, USA) after 10 min of exposure to the growth light.

The plant height was measured by a ruler from the collar (the point on the stem where the roots start to grow) to the leaf base of the highest fully expanded leaf.

The measurements of the leaf area were obtained as follows. The leaves were separated from the plants and photographed at a 90 degree angle to the leaf surface (Canon IXUS 220HS, Monterey, CA, USA). The images were then analyzed using LIA for Win32 software to obtain the leaf area.

The determination of the fresh and dry weight was achieved as follows. The plants from each glass culture vessel were separated and weighed for their fresh weight using a HR-202i balance (A&D Company, Limited, Japan). For the dry weight determination, the plants were dried in an electric drying oven (UNB 500, Memmert, Germany) at 60 °C for three days until a constant mass was achieved.

### 4.4. Determination of the Triterpenoid Content

The *H. corymbosa* extraction samples from the different LED light treatments were obtained following the method of Li et al. [48]. Briefly, the dry powder sample (1 g) was weighed and extracted with 20 mL of 70% ethanol and the microwave method was used (Panasonic, auto sensor diet, and full power) at 60 °C. The supernatant was then collected by centrifuging (6000 rpm/min) and vacuumed to dryness. The total triterpenoid content was determined by the colorimetric reaction method. Vanillin and perchloric acid were used as an oxidant; the distinctive color of this reaction is purple [49]. The dried extraction sample was dissolved in 14 mL of 95% methanol; 1 mL of the sample solution was heated in a 5 mL volumetric flask to evaporation in a 70 °C water bath and 0.6 mL of a new mixed 5% (*w/v*) vanillin-anhydrous (water-free) acetic acid solution and 1.2 mL perchloric acid were added and mixed. The reaction solution was heated at 70 °C for 20 min to allow full-color development and then cooled in ice water. The solution volume was then adjusted to 5 mL using acetic acid. The total triterpenoid content was calculated as mg ursolic acid equivalents (UAE) per gram dry weight by optical density at 550 nm (GENESYS™ 30, Thermo Fisher Scientific Inc., USA) and the ursolic acid standard curve. The blank consisted of all reagents and solvents without a sample solution.

### 4.5. Statistical Analysis

The average data of three subsamples/glass culture vessels were used as a replication. All experiments were conducted with five glass culture vessels per LED treatment. The data analysis was performed by R software (Ver. 4.1.2, the R foundation for statistical computing, Vienna, Austria). The dataset did not satisfy normality; thus, the non-parametric data analysis Kruskal–Wallis test was used. For the post hoc test, the Dunn test with the Benjamini–Hochberg correction was applied. At the fresh weight result, the Kruskal–Wallis test *p*-value was lower than 0.05; however, the post hoc test represented no difference between the treatments. The box plots were produced using the ‘ggpubr’ package in R 4.1.2.

## 5. Conclusions

Blue and red LEDs caused an antagonistic trend in the photosynthesis and photomorphogenesis of *H. corymbosa*. Being exposed to a high proportion of red LEDs (i.e., R and B3R7) negatively affected the plant photochemical efficiency (Y(II) and qP) and also led to a decrease in the Hill reaction activity. A high proportion of 450 nm blue LEDs, on the other hand, did not reduce the F_v_/F_m_, Y(II), and Y(NO) values among the FL, B, B3R7, and B5R5 treatments. Therefore, the plants under high blue/red LED ratios were more suitable for plant biomass accumulation compared with those grown under high red/blue LED ratios. Furthermore, monochromatic blue LED lights played an important role in the total triterpenoid production in the leaves of *H. corymbosa*.

## Figures and Tables

**Table 1:** The fluorescence parameters in leaves sampled from *H. corymbosa* plantlets grown in vitro under different ratios of red and blue light at a 50 μmol·m^−2^·s^−1^ light intensity.

Light Source	F_v_/F_m_	Y(II)	Y(NPQ)	Y(NO)	qP	qN	ETR (μmol Electron·m^−2^·s^−1^)
FL	0.749 ^a^	0.666 ^a^	0. 037 ^a^	0.312 ^b^	0.801 ^bc^	0.311 ^a^	12.179 ^ab^
B	0.753 ^a^	0.623 ^a^	0.075 ^a^	0.302 ^b^	0.875 ^ab^	0.273 ^a^	13.259 ^a^
B7R3	0.739 ^ab^	0.663 ^a^	0.043 ^a^	0.289 ^b^	0.957 ^a^	0.154 ^a^	14.159 ^a^
B5R5	0.737 ^ab^	0.662 ^a^	0.028 ^a^	0.309 ^b^	0.924 ^ab^	0.147 ^a^	13.902 ^a^
B3R7	0.681 ^b^	0.510 ^b^	0.074 ^a^	0.360 ^a^	0.768 ^bc^	0.271 ^a^	9.849 ^b^
R	0.694 ^b^	0.420 ^b^	0.095 ^a^	0.396 ^a^	0.734 ^c^	0.267 ^a^	8.978 ^b^

Statistical analysis was performed using a Kruskal–Wallis test (*p*-value ≤ 0.05) followed by a Dunn–Benjamini–Hochberg post hoc test for multiple comparisons. Different letters indicate a significant difference between two medians.

**Table 2:** SPAD value and Hill reaction activity of *H. corymbosa* leaves after 4 weeks of growth under different light sources at a 50 μmol·m^−2^·s^−1^ light intensity.

Light Source	SPAD Value	Hill Reaction Activity (nmol DCIP.Million of Chloroplast^−1^·min^−1^)
FL	26.2 ^a^	0.087 ^a^
B	23.9 ^b^	0.085 ^ab^
R	19.7 ^c^	0.076 ^b^

Statistical analysis was performed with a Kruskal–Wallis Test (*p*-value ≤ 0.05) followed by a Dunn–Benjamini–Hochberg post hoc test for multiple comparisons. Different letters indicate a significant difference between two medians.

**Table 3:** Total triterpenoid content of *H. corymbosa* leaves after 4 weeks of growth under different light sources at the same light intensity of 50 µmol·m^−2^·s^−1^.

Light Source	Total Triterpenoid (mg _UAE_/g _DW_)
FL	27.03 ^a^
B	26.33 ^a^
R	18.41 ^b^

Statistical analysis was performed with a Kruskal–Wallis Test (*p*-value ≤ 0.05) followed by a Dunn–Benjamini–Hochberg post hoc test for multiple comparisons. Different letters indicate a significant difference between two medians.

## Data Availability

The data presented in this study are available on request from the corresponding author. The data are not publicly available due to privacy.
